# The presence of leukoaraiosis enhances the association between sTWEAK and hemorrhagic transformation

**DOI:** 10.1002/acn3.51171

**Published:** 2020-10-06

**Authors:** Andrés da Silva‐Candal, María Pérez‐Mato, Manuel Rodríguez‐Yáñez, Iria López‐Dequidt, José M. Pumar, Paulo Ávila‐Gómez, Tomás Sobrino, Francisco Campos, José Castillo, Pablo Hervella, Ramón Iglesias‐Rey

**Affiliations:** ^1^ Clinical Neurosciences Research Laboratory (LINC) Health Research Institute of Santiago de Compostela (IDIS) Santiago de Compostela Spain; ^2^ Neuroscience and Cerebrovascular Research Laboratory La Paz University Hospital IdiPAZ UAM Paseo de la Castellana 261 Madrid 28046 Spain; ^3^ Stroke Unit Department of Neurology Hospital Clínico Universitario Santiago de Compostela Spain; ^4^ Department of Neuroradiology Hospital Clínico Universitario Health Research Institute of Santiago de Compostela (IDIS) Santiago de Compostela Spain

## Abstract

**Objective:**

To investigate whether elevated serum levels of sTWEAK (soluble tumor necrosis factor‐like inducer of apoptosis) might be involved in a higher frequency of symptomatic hemorrhagic transformation (HT) through the presence of leukoaraiosis (LA) in patients with acute ischemic stroke (IS) undergoing reperfusion therapies.

**Methods:**

This is a retrospective observational study. The primary endpoint was to study the sTWEAK‐LA‐HT relationship by comparing results with biomarkers associated to HT and evaluating functional outcome at 3‐months. Clinical factors, neuroimaging variables and biomarkers associated to inflammation, endothelial/atrial dysfunction or blood‐brain barrier damage were also investigated.

**Results:**

We enrolled 875 patients (mean age 72.3 ± 12.2 years; 46.0% women); 710 individuals underwent intravenous thrombolysis, 87 endovascular therapy and 78 both. HT incidence was 32%; LA presence was 75.4%. Patients with poor functional outcome at 3‐months showed higher sTWEAK levels at admission (9844.2 [7460.4–12,542.0] vs. 2717.3 [1489.7–5852.3] pg/mL, *P* < 0.0001). By means of logistic regression models, PDGF‐CC and sTWEAK were associated with mechanisms linked simultaneously to HT and LA. Serum sTWEAK levels at admission ≥6700 pg/mL were associated with an odds ratio of 13 for poor outcome at 3‐months (OR: 13.6; CI 95%: 8.2–22.6, *P* < 0.0001).

**Conclusions:**

Higher sTWEAK levels are independently associated with HT and poor functional outcome in patients with IS undergoing reperfusion therapies through the presence of LA. sTWEAK could become a therapeutic target to reduce HT incidence in patients with IS.

## Introduction

Hemorrhagic transformation (HT), which refers to a spectrum of ischemia‐related brain hemorrhage, is a frequent spontaneous complication of ischemic stroke (IS), especially after reperfusion treatments (intravenous or intraarterial fibrinolysis, thrombectomy, or both procedures).^1^, ^2^ The incidence of spontaneous HT ranges from 38% to 71% in autopsy studies and from 13% to 43% in CT/MRI studies, whereas the incidence of symptomatic HT is between 1% and 20%.^3^, ^4^, ^5^, ^6^ Therefore, HT is a dreaded complication that limits the use of reperfusion treatment, the only method of clinical management of acute IS, thereby leading to poor long‐term function outcome.

Previous research has identified several independent predictors of symptomatic HT in patients with IS; these risk factors include age, cardioembolism, thrombolysis, large lesions, history of hypertension and BP, and blood glucose on admission.^5^, ^7^ Moreover, potential biomarkers for predicting HT in patients with IS have been proposed, specifically those related to: (1) disruption of the blood brain barrier (BBB); (2) inflammation and oxidative stress; (3) vascular endothelial growth factor (VEGF) and other angiogenesis factors; and (4) coagulation/ fibrinolysis disorder.^8^

On the other hand, cerebral microbleeds and the presence of leukoaraiosis (LA) or white matter lesions have been linked to symptomatic HT and poor functional outcome. LA is a gradual disease affected by different risk factors. This suggests that the degrees of LA may indicate several pathogenesis and influence differentially the prognosis of the patients. In this line, there are clinical evidences that moderate to severe LA presence may be related to endothelial dysfunction and BBB damage.^9^, ^10^, ^11^, ^12^

We have recently identified an endothelial dysfunction marker, the soluble tumor necrosis factor‐like inducer of apoptosis (sTWEAK), as a possible biomarker related to LA presence in patients with IS.^13^ sTWEAK is constitutively expressed by monocytes, tumor cell lines, and endothelial cells. It acts on responsive cells by binding to its receptor on cell surface (fibroblast growth factor‐inducible 14 [Fn14]), and then activates multiple pro‐inflammatory molecules. Preclinical studies have also demonstrated that the sTWEAK‐Fn14 pathway is involved in a considerable number of pathologies and could have important implications for the progression of intracerebral hemorrhage.^14^, ^15^, ^16^, ^17^

At present, evaluating the risk of symptomatic HT in patients with IS mainly depends on clinical status and neuroimaging studies. Further studies, therefore, are needed to identify risk factors and clinical or biological markers that could become therapeutic targets for HT prevention. We hypothesized that elevated serum levels of sTWEAK might be involved in a higher frequency of symptomatic HT through the presence of LA. In the present study, we intend to investigate the possible relationship among sTWEAK – LA – symptomatic HT in IS individuals undergoing reperfusion therapies; compare results with other biomarkers associated to symptomatic HT and evaluate the functional outcome at 3 months.

## Material and Methods

### Patient screening

We conducted a retrospective observational study of patients with ischemic stroke admitted to the Stroke Unit of the Hospital Clínico Universitario of Santiago de Compostela (Spain), who were prospectively registered in an approved data bank (BICHUS). All patients were treated by a certified neurologist according to national and international guidelines.^18^ Inclusion criteria for this analysis were the following: (1) authorization for the anonymous use of individuals’ data for research purposes; (2) neuroimaging study (MRI or CT study at inclusion and between 4th–7th day); (3) hemispheric location lesion; (4) patients undergoing reperfusion therapy; 5) known stroke onset time, with a latency time between stroke onset and inclusion ≤6 h; (6) follow‐up (personal interview or telephone contact) at 3 months; (7) patients without previous comorbidity, and a life expectancy of less than half a year; (8) blood sample available in the stroke biobank. Data for this study were retrospectively analyzed, the time frame was between January 2008 and December 2018.

This research was carried out in accordance with the Declaration of Helsinki of the World Medical Association (2008) and approved by the Ethics Committee of Santiago de Compostela (2019/616). All patients or their relatives signed the informed consent for inclusion in the registry and for anonymous use of their personal data for research purposes.

### Demographic variables

The demographic variables evaluated in the present study were: age, gender, history of high blood pressure (at least 2 blood pressure measurements >140/85 mmHg or under antihypertensive treatment), diabetes (previous diagnosis or under anti‐diabetic treatment), smoking (habitual smoker or up to the previous year), alcohol consumption (>300 g of alcohol per week), dyslipidemia (at least a previous measurement of total cholesterol >230 mg/dL or lipid lowering therapy), peripheral arterial disease, coronary disease, atrial fibrillation, heart failure, previous stroke, prior transient ischemic attack (TIA), and treatment with anticoagulants and antiplatelets.

### Clinical variables

Patients’ clinical situation was evaluated by a certified neurologist using the National Institute of Health Stroke Scale (NIHSS)^19^ at admission, every 6 h during the first day, and every 24 h during hospitalization. The modified Rankin Scale (mRS)^20^ was used to evaluate functional outcome at discharge and at 3 months. Effective reperfusion was defined as ≤8 points in the NIHSS during the first 24 h. Early neurological deterioration was defined as an increase of ≥4 points in the NIHSS within the first 48 h. An mRS score >2 at 3 months was considered a poor functional outcome. Finally, the registry includes time from stroke onset to reperfusion therapies and maximum axillary temperature during the first 24 h.

### Neuroimaging studies

Neuroimaging studies were analyzed by a neuro‐radiologist supervised by the above certified neurologist. The identification of symptomatic hemorrhagic transformation, defined according to ECASS II criteria,^21^ was performed at the time of recording the neurological worsening (minor symptomatic: >2–3 point increase in the NIHSS; major symptomatic: ≥4 point increase in the NIHSS), and by means of a second CT performed between the 4th–7th day. The lesion volume was calculated in the CT performed between the 4th–7th day using ABC/2 method.^22^ The presence and intensity of leukoaraiosis were assessed using the Fazekas scale^23^ with a total score of 0 to 6 (group I, 1–2; group II, 3–4; group III, 5–6), and were identified in the neuroimaging performed at admission. Stroke diagnosis was made using the TOAST criteria.^24^

### Biomarkers

In our study, blood samples were measured for glucose, glycosylated hemoglobin, erythrocyte sedimentation rate (ESR), triglycerides, LDL and HDL cholesterol. We selected soluble tumor necrosis factor‐like inducer of apoptosis (sTWEAK) as a possible biomarker associated with endothelial dysfunction and rupture of the blood‐brain barrier (BBB). These are mechanisms that are considered to be close to LA development.^14^, ^15^, ^16^, ^17^ We compared the results of sTWEAK with other potential biomarkers for predicting hemorrhagic transformation of ischemic stroke^8^; those related to inflammation and oxidative stress processes namely leukocytes, fibrinogen, C‐reactive protein, erythrocyte sedimentation rate, serum levels of interleukin 6 (IL‐6), and tumor necrosis factor alpha (TNF*α*)^25^, ^26^; those correlating with BBB disruption such as microalbuminuria, matrix metalloproteinase‐9 (MMP‐9), serum levels of S100‐*β*, neuron‐specific enolase (NSE), and neuroserpin^27^, ^28^; those involved in vasoreactivity including cell fibronectin (c‐Fn), endothelin‐1 (ET‐1), and platelet‐derived growth factor‐CC isoform (PDGF‐CC)^29^, ^30^; and finally, those related to serum levels of glutamate were determined as an excitotoxicity marker.^31^

Blood samples to measure biomarkers were obtained before administering the reperfusion treatment. Biomarkers were evaluated within the 3 months following blood sample collection. Biochemistry, hematology, and coagulation tests were assessed in the central laboratory of the Hospital Clínico Universitario of Santiago de Compostela – blinded to clinical and neuroimaging data. IL‐6, sTWEAK, TNF*α*,ET‐1, c‐Fn MMP‐9, neuroserpin, NSE, S100‐*β*, PDGF‐CC, and glutamate measurements were performed at the Clinical Neurosciences Research Laboratory by researchers blinded to clinical and neuroimaging data.

IL‐6 was measured from ELISA kit (BioLegend, San Diego, USA). sTWEAK was measured using Human TWEAK ELISA Kit (Elabscience, Texas, USA), and TNF*α* was measured using an immunodiagnostic IMMULITE 1000 System (Siemens Healthcare Global, Los Angeles, USA). Minimum assay sensitivity was 1.6 pg/mL, 4.69 pg/mL and 1.7 pg/mL respectively with an intra‐ and inter‐assay coefficient of variation (CV) <10%. Serum levels of ET‐1, c‐Fn, and MMP‐9 were measured with commercially available quantitative sandwich enzyme‐linked immunoabsorbent assay kits obtained from Biomedica Medizinprodukte GMBH (Vienna, Austria); Biohit Plc (Milan, Italy); and Biotrack, Amersham Pharmacia (Uppsala, Sweden) respectively. Each sample was assayed in duplicate and inter‐/intra‐assay CV were always <10%. For neuroserpin quantification, a sandwich ELISA (Assay Biotechnology, CA, USA) was performed as described previously.^27^ Each sample was assayed in duplicate and inter‐/intra‐assay CV were always <15%. Serum NSE and S100‐*β* levels were measured with an electrochemiluminescence immunoassay using the ELECSYS 2010 (Roche Diagnostics, Penzberg, Germany). Sensitivity of the method is 0.05 ng/mL for NSE and 0.005 mg/L for S100‐*β*. The interassay CV was 4.2% for NSE and 2.4% for S100‐*β* measurements. Serum levels of PDGF‐CC were quantified by using an antibody capture ELISA which is specific for cleaved and active growth factor (ZymoGenetics, Inc., Seattle, USA) as described before.^29^ Serum glutamate level was determined by high performance liquid chromatography (1260 Infinity II, Agilent Technologies, Santa Clara, CA, USA) using the AccQ‐Tag™ Precolumn derivatization method for amino acid analysis (Waters, Milford, MA, USA), following a previously described method.^32^

### Endpoints

The primary endpoint of this study was to analyze the relationship between serum levels of sTWEAK, LA, and symptomatic HT. The relationship with other biomarkers associated to symptomatic HT (IL‐6, TNF*α*, MMP‐9, S100‐*β*, NSE, neuroserpin, c‐Fn, ET‐1, PDGF‐CC, and glutamate), and the functional outcome at 3 months were evaluated as secondary endpoints.

### Statistical analysis and sample size calculation

Firstly, a descriptive analysis was performed. Categorical variables were described with frequency and percentage. The continuous variables were described with mean and standard deviation or median and interquartile range, depending on their adjustment to a normal distribution (which was determined with the Kolmogorov–Smirnov test with the Lilliefors correction). Then, statistical inference was carried out with the chi‐square test, *t*‐student, or Mann–Whitney according to the nature of the contrast variable and its adjustment to normality. Finally, a multivariate logistic regression was performed with the significant variables found in the previous analysis. The results were expressed as odds ratio (OR) with 95% confident intervals (95% CI). Moreover, in order to detect the ability of biomarkers to classify the values associated with HT, ROC (Receiver Operating Characteristic) curves were developed, converting continuous variables into categorical ones for a value that offers maximum sensitivity and specificity. Significant values of *P* < 0.05 were considered. All analyses were performed with IBM SPSS Statistics v.20.

For this analysis, we consecutively screened all the patients who met all the inclusion criteria from December 2018 backwards. To achieve a power of 80% to detect differences in the contrast of the null hypothesis H0 (the difference in means is equal to the limit of superiority, by means of a unilateral t‐student test –of superiority‐ for two independent samples) taking into account that the level of significance is 5%, and assuming that the limit of superiority is 2000.00 pg/mL, the average of sTWEAK in the group of good outcome is 2035.50 pg/mL whereas in the poor outcome it is 4150.40 pg/mL. The standard deviation of both groups was approximately 500.00 pg/mL. In addition, the proportion of poor outcome for our cohort was estimated at 30%. Therefore, at least 168 individuals with an unfavorable outcome and 392 with a favorable outcome should be enrolled. In addition, 10 more cases should be enrolled for each adjustment covariate in the multivariate analysis (3‐4 of adverse outcome and 6‐7 of favorable outcome, depending on the justified proportion). Assuming the need to adjust the model for 220 covariables, at least 248 cases with a poor outcome and 497 with a good outcome are needed. The calculation was made using the program Ene 3.0.

### Data availability statement

All data are available within the text of the manuscript. Further anonymized data could be made available to qualified investigators upon reasonable request.

## Results

### Sample description

A total of 875 patients with IS were enrolled during the study period (54.0% males; mean age 69.6 ± 11.8 years vs. 46.0% females; mean age 74.9 ± 12.6 years). Of these, 710 individuals underwent intravenous thrombolysis, 87 endovascular therapy (intraarterial thrombolysis or mechanical thrombectomy) and 78 both intravenous and endovascular therapy. According to the TOAST classification, 206 individuals were classified as atherothrombotic (23.5%), 381 as cardioembolic (43.5%), 11 as lacunar (1.3%), and 277 as undetermined (31.7%). The average time between the detection of symptoms and the administration of the treatment was 161.8 ± 61.2 min.

Tables 1 and 2 detail the baseline demographic variables, clinical characteristics, neuroimaging parameters, and molecular markers per functional outcome groups. A good outcome was observed in 620 individuals (70.9%) and an adverse outcome was observed in 255 (29.1%).

**Table 1:** Univariate analysis. Baseline demographic data, clinical characteristics, and neuroimaging variables in IS patients with good or poor outcome at 3 months (*n* = 875).

	Good outcome (*n* = 620)	Poor outcome (*n* = 255)	*P* value
Demographic variables			
Age, years	71.2 ± 12.6	74.1 ± 12.0	0.002
Female gender, %	41.8	56.1	<0.0001
Arterial hypertension, %	63.3	65.1	0.643
Diabetes, %	22.1	24.7	0.426
Smoking, %	17.6	10.6	0.010
Alcohol consumption, %	10.5	9.8	0.808
Hyperlipidemia, %	38.9	40.4	0.704
Peripheral arterial disease, %	6.5	7.5	0.656
Ischemic heart disease, %	12.1	14.1	0.434
Atrial fibrillation, %	20.6	28.2	0.017
Heart failure, %	4.0	5.1	0.470
Previous stroke, %	13.1	14.9	0.759
Previous transient ischemic attack, %	7.4	4.7	0.178
Previous platelet antiaggregants, %	28.2	28.6	0.934
Previous anticoagulants, %	7.7	9.4	0.419
Clinical variables			
Previous mRS	0 [0, 0]	0 [0, 0]	0.245
NIHSS at admission	16 [12, 20]	19 [15, 23]	<0.0001
NIHSS at admission–NIHSS at 24 h	8.6 ± 7.5	−0.2 ± 5.6	<0.0001
≤8 points of the NIHSS in 24 h, %	58.1	9.8	<0.0001
Early neurological deterioration, %	2.7	28.8	<0.0001
Onset‐treatment time, min	154.1 ± 59.8	180.6 ± 60.4	<0.0001
Maximum axillary temperature 24 h, °C	36.2 ± 0.6	36.8 ± 0.8	<0.0001
Neuroimaging variables			
Volume of ischemic lesion, mL	25.1 ± 41.2	114.3 ± 99.9	<0.0001
Leukoaraiosis degree			<0.0001
No, %	55.7	7.9	
Grade I, %	41.6	23.0	
Grade II, %	2.7	44.7	
Grade III, %	0	23.5	
Hemorrhagic transformation			<0.0001
NO, %	67.7	68.5	
IH1, %	25.9	10.6	
IH2, %	4.5	7.1	
PH1, %	1.3	7.1	
PH2, %	0.6	6.7	
TOAST criteria			0.001
Atherothrombotic, %	23.7	23.1	
Cardioembolic, %	40.0	52.2	
Lacunar, %	1.8	0	
Indeterminate, %	34.5	24.7	

**Table 2:** Univariate analysis. Molecular markers and HT biomarkers findings in IS patients with good or poor outcome at 3 months (*n* = 875).

	Good outcome (*n* = 620)	Poor outcome (*n* = 255)	*P* value
Molecular markers			
Blood glucose, mg/dL	133.7 ± 53.7	150.7 ± 58.6	<0.0001
Glycosylated hemoglobin, %	6.3 ± 5.2	5.9 ± 1.3	0.364
Erythrocyte sedimentation rate, mm	17.4 ± 19.9	20.6 ± 21.6	0.060
LDL cholesterol, mg/dL	108.4 ± 41.6	107.0 ± 38.5	0.723
HDL cholesterol, mg/dL	40.9 ± 114.2	43.4 ± 16.6	0.056
Triglycerides, mg/dL	112.9 ± 49.1	117.3 ± 56.9	0.320
Leukocytes at admission, ×103/mL	7.8 ± 2.9	9.4 ± 3.5	<0.0001
Fibrinogen at admission, mg/dL	402.8 ± 101.5	441.8 ± 102.3	<0.0001
C‐reactive protein at admission, mg/L	3.2 ± 3.9	6.1 ± 4.6	<0.0001
Platelets, ×103/mL	201.7 ± 67.5	206.7 ± 65.6	0.311
Miocroalbuminuria, mg/24 h	5.0 ± 7.1	7.3 ± 9.2	<0.0001
HT biomarkers			
Glutamate, μmol/L/mL	98.9 ± 55.2	131.7 ± 59.9	<0.0001
TNF‐μ, pg/mL	14.4 ± 5.5	15.8 ± 5.8	0.001
IL‐6, pg/mL	6.3 [2.3–11.6]	8.5 [4.6–13.6]	<0.0001
Cellular fibronectin, mg/L	2.2 [1.6–2.7]	2.1 [1.5–2.9]	0.797
Metalloproteinase‐9, ng/mL	51.2 [29.4–85.2]	58.4 [33.0–97.4]	0.121
Endothelin‐1, fmol/mL	1.5 [0.9–3.6]	1.4 [ 0.7–2.9]	0.193
Neuron‐specific enolase, ng/mL	7.4 ± 1.6	8.8 ± 1.7	<0.0001
S100‐*β*, μg/mL	0.07 [0.05–0.09]	0.14 [0.08–0.16]	<0.0001
Neuroserpin, ng/mL	248.9 [226.4–267.2]	241.2 [204.8–269.6]	0.217
PDGF‐CC, ng/mL	73.1 ± 13.7	86.5 ± 13.5	<0.0001
sTWEAK, pg/mL	2717.3 [1489.7–5852.3]	9844.2 [7460.4–12542.0]	<0.0001

According to demographic and clinical variables, patients with unfavorable outcome were older (74.1 ± 12 years vs. 71.2 ± 12.6 years, *P* = 0.002), more frequently women (41.8% vs. 56.1, *P* < 0.0001), showed stronger smoking habits, and suffered from atrial fibrillation (20.6% vs. 28.2%, *P* = 0.017). Moreover, they had higher neurological deficits evaluated by NIHSS at admission (19 [15, 23] vs. 16 [12, 20], *P < *0.0001), lower rate of neurological improvement [ΔNIHSS ≤ 8 points in the first 24 h] (−0.2 ± 5.6 vs. 8.6 ± 7.5, *P < *0.0001), lower effective reperfusion (9.8% vs. 58.1%, *P* < 0.0001), higher early neurological deterioration (28.8% vs. 2.7%, *P* < 0.0001), longer onset‐treatment time (180.6 ± 60.4 min vs. 154.1 ± 59.8 min, *P* < 0.0001), and higher maximum axillary temperature at admission (36.8 ± 0.8°C vs. 36.2 ± 0.6°C, *P* < 0.0001).

Furthermore, patients with poor functional outcome showed higher lesion volume (114.3 ± 99.9 mL vs. 25.1 ± 41.2 mL, *P* < 0.0001). There were also significant differences between good and poor outcome individuals for the presence and degree of LA, symptomatic HT and TOAST criteria (Table 1).

### Relationship between symptomatic HT and outcome

**Figure 1:**
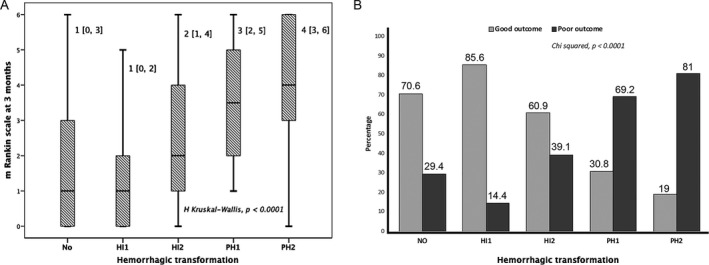
(A) Association between mRS score at 3 months and HT subtype. (B) Relationship between HT subtype and functional outcome at 3 months.

Symptomatic HT was noted in 280 (32%) patients; of these patients, an unfavorable outcome was observed in 80 (28.6%). By contrast, no symptomatic HT was found in 595 (68%) individuals; and surprisingly similar rates for poor functional outcome were observed 29.4% (175 patients). Figure 1A and B detail the correlation between mRS score at 3 months and HT subtype, as well as the description of the patients' functional outcome. They show that the IH1 subtype was related to functional outcome improvement, but PH1 and PH2 were associated with an unsatisfactory outcome at 3 months.

### Relationship between symptomatic HT and effective reperfusion

**Figure 2:**
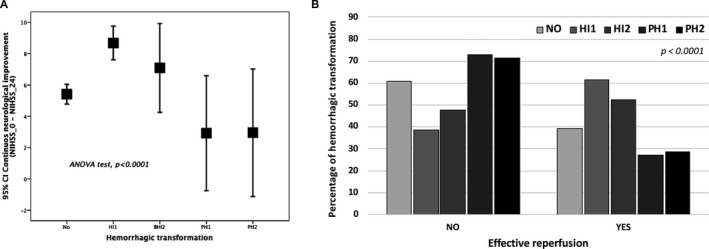
(A) Relationship between neurological improvement and HT subtype. (B) Relationship between effective reperfusion and the presence/degree of HT.

Figure 2A and B show the association between effective reperfusion and the presence/ degree of symptomatic HT. Of the patients with HT, 152 (54.3%) had effective reperfusion, in contrast with 128 (45.7%) without effective reperfusion. This result is different for patients without HT; 362 (60.8%) without effective reperfusion versus 233 (39.2%) with effective reperfusion. Among effective reperfusion individuals, IH1 and IH2 were more frequent; on the contrary PH1 and PH2 were more common in patients without effective reperfusion.

### Relationship between sTWEAK levels, LA and HT in patients with poor functional outcome

**Figure 3:**
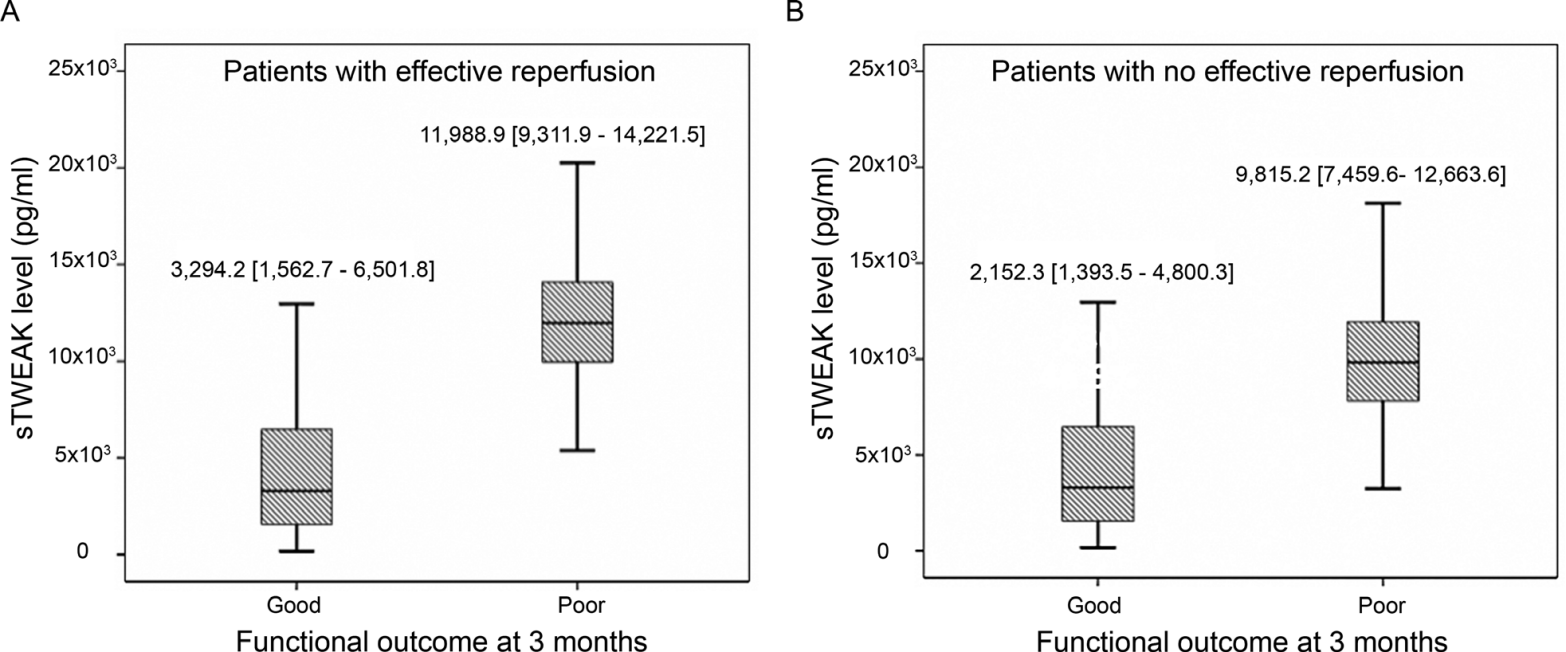
Relationship between sTWEAK levels and functional outcome at 3 months in patients with effective reperfusion (A) and without effective reperfusion (B).

We found that patients with poor functional outcome had higher levels of sTWEAK at admission (9844.2 [7460.4–12,542.0] pg/mL vs. 2717.3 [1489.7–5852.3] pg/mL, *P* < 0.0001). Effective reperfusion was observed in 385 (44%) patients, of whom 360 (93.5%) showed a high frequency of good functional outcome. No effective reperfusion was detected in 490 (56%) individuals, of whom 53.1% (260 patients) showed a lower frequency of good functional outcome. However, in both cases, higher sTWEAK levels were associated with a poor functional outcome at 3 months (Fig. 3).

### Relationship between LA and symptomatic HT

**Figure 4:**
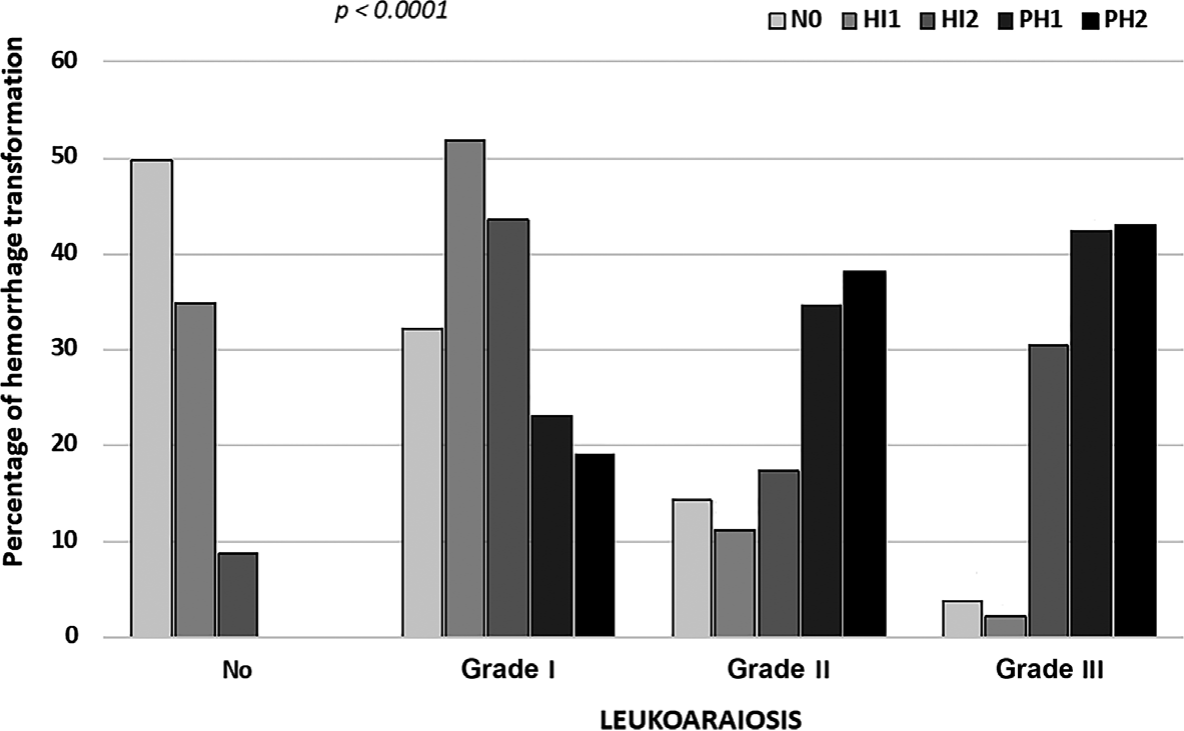
Correlation between the presence/degree of LA and HT.

It is interesting to note that the presence of LA worsens functional patient outcome proportionally to LA grade. The presence and degree of LA had a significant impact (*P* < 0.0001) on symptomatic HT in patients with IS, as illustrated in Figure 4. Among 280 individuals with HT, the presence of LA was found in 211 (75.4%) patients, and in 69 (24.6%) the presence of LA was not noted. On the other hand, of 595 individuals with no HT, LA was observed in 299 (50.3%), and 296 (49.7%) did not show the presence of LA.

By means of logistic regression models adjusted for those significant demographic, clinical, neuroimaging, and molecular variables of bivariate analysis showed in Tables 1 and 2, we determined that LA and symptomatic HT were interrelated variables as HT subtypes IH2, PH1 and PH2 were associated with poor functional outcome. For this reason, LA and HT were not be included in the same model. In this regard, two independent logistic regression analysis with symptomatic HT subtypes or LA grades showed that female gender (OR: 2.51; CI 95%: 1.29–4.92; *P* = 0.007), atrial fibrillation (OR: 1.79; CI 95%: 1.29–4.92; *P* = 0.007), NIHSS at admission (OR: 1.09; CI 95%: 1.02–1.16; *P* < 0.0001), effective reperfusion (OR: 0.08; CI 95%: 0.31–0.20; *P* < 0.0001), maximum axillary temperature (OR: 2.01; CI 95%: 1.01–3.16; *P* = 0.045), ischemic lesion volume (OR: 1.01; CI 95%: 1.01–1.02; *P* < 0.0001), symptomatic HT subtypes, LA degree, and C‐ reactive protein (OR: 1.08; CI 95%: 1.02–1.14; *P* = 0.011) were independently associated with poor outcome at 3 months (Tables 3 and 4).

**Table 3:** Logistic regression analysis including demographic, clinical, neuroimaging, molecular variables, and LA degree. Dependent variable: Poor outcome at 3 months.

Independent variables	Not adjusted			Adjusted		
	OR	CI 95%	*P* value	OR	CI 95%	*P* value
Age	1.02	1.01–1.03	0.002	1.01	0.99–1.03	0.280
Female gender	1.78	1.33–2.29	<0.0001	2.4	1.45–3.99	0.001
Latency time	1.01	1.01–1.01	<0.0001	1.00	1.00–1.01	0.201
Smoker	0.56	0.35–0.87	0.010	0.56	0.26–1.20	0.134
Atrial fibrillation	1.51	1.08–2.11	0.015	1.79	1.01–3.16	0.045
Temperature	3.06	2.43–3.86	<0.0001	1.55	1.07–2.24	0.019
Blood glucose	1.01	1.00–1.01	<0.0001	1.00	1.00–1.09	0.270
Leukocytes	1.16	1.11–1.21	<0.0001	1.00	0.93–1.09	0.916
Microalbuminuria	1.04	1.02–1.06	<0.0001	0.98	0.95–1.02	0.247
C‐reactive protein	1.17	1.12–1.21	<0.0001	1.08	1.02–1.14	0.011
NIHSS at admission	1.08	1.05–1.11	<0.0001	1.12	1.06–1.17	<0.0001
Lesion volume	1.02	1.02–1.02	<0.0001	1.01	1.01–1.02	<0.0001
≤8 points NIHSS 24 h	0.08	0.05–0.12	<0.0001	0.05	0.02−0.10	<0.0001
Hemorrhagic transformation						
IH1	0.41	0.26–0.63	<0.0001	0.27	0.12–0.59	0.001
IH2	1.54	0.83–2.86	0.169	0.80	0.23–2.78	0.729
PH1	5.4	2.31–12.65	<0.0001	4.99	1.22–20.40	0.025
PH2	10.2	3.38–30.75	<0.0001	12.19	1.17 −91.8	0.015

**Table 4:** Logistic regression analysis including demographic, clinical, neuroimaging, molecular variables, and HT subtype. Dependent variable: Poor outcome at 3 months.

Independent variables	Not adjusted			Adjusted		
	OR	CI 95%	*P* value	OR	CI 95%	*P* value
Age	1.02	1.01–1.03	0.002	1.02	0.99–1.05	0.235
Female gender	1.78	1.33–2.29	<0.0001	2.51	1.29–4.92	0.007
Latency time	1.01	1.00–1.01	<0.0001	1.00	0.99–1.01	0.158
Smoker	0.56	0.35–0.88	0.010	0.81	0.29–2.36	0.697
Atrial fibrillation	1.51	1.08–2.11	0.015	2.01	0.94–4.29	0.072
Temperature	3.06	2.43–3.86	<0.0001	1.85	1.14–2.99	0.013
Blood glucose	1.01	1.00–1.01	<0.0001	1.00	1.00–1.01	0.482
Leukocytes	1.16	1.11–1.22	<0.0001	0.96	0.86–1.07	0.490
Microalbuminuria	1.04	1.02–1.06	<0.0001	0.97	0.93–1.01	0.126
C‐reactive protein	1.16	1.19–1.21	<0.0001	1.05	0.97–1.14	0.217
NIHSS at admission	1.08	1.05–1.11	<0.0001	1.09	1.02–1.16	0.009
Lesion volume	1.02	1.02–1.03	<0.0001	1.01	1.01–1.02	<0.0001
≤8 points NIHSS 24 h	0.08	0.5–0.12	<0.0001	0.08	0.31–0.20	<0.0001
Leukoaraiosis degree						
Grade I	4.08	2.4–6.93	<0.0001	4.01	1.90–8.48	<0.0001
Grade II, III	175.56	90.19−345.64	<0.0001	175.64	65.55–470.62	<0.0001

### Secondary endpoints: relationship of sTWEAK and other biomarkers associated with symptomatic HT and LA with poor functional outcome

Analysis of molecular markers showed that patients with unfavorable outcome had higher levels of blood glucose, inflammation markers (leukocytes, fibrinogen, C‐reactive protein), and miocroalbuminuria as endothelial dysfunction marker, where the high levels of fibrinogen (441.8 ± 102.3 mg/dL vs. 402.8 ± 101.5 mg/dL; *P* < 0.0001) and C‐reactive protein (6.1 ± 4.6 mg/L vs. 3.2 ± 3.9 mg/L; *P* < 0.0001) should be highlighted (Table 2).

HT biomarkers indicated that, in addition to sTWEAK, high levels of glutamate, TNF*α*, IL‐6, NSE, S100‐*β*, and PDGF‐CC were more frequent in patients with unfavorable outcome, where the evident difference in glutamate level (131.7 ± 59.9 μmol/L per mL vs. 98.9 ± 55.2 μmol/L per mL, *P* < 0.0001), and S100‐*β* (0.14 [0.08–0.16] *μ*g/mL vs. 0.07 [0.05–0.09] *μ*g/mL) is worthy of attention.

With the exception of TNF‐*α*, NSE, and S100‐β, all biomarkers used in this analysis were associated with symptomatic HT and its severity (*P* < 0.0001) (Fig. S1). Besides, with the exception of neuroserpin, all biomarkers used in this analysis were correlated with the presence and degree of LA (*P* < 0.0001) (Fig. S2).

ROC curve analysis of glutamate, TNF*α*, IL‐6, NSE, S100‐*β*, PDGF‐CC, and sTWEAK levels for the functional outcome at 3 months revealed a high average area under the curve (0.72 ± 0.11). In particular, ROC analysis showed that sTWEAK levels at admission ≥6700 pg/mL suggested unfavorable outcome at 3 months with a specificity of 82% and sensitivity of 81% (area under the curve: 0.883; 95% CI: 0.860–0.905; *P* < 0.0001). Similarly, S100‐*β* levels ≥ 0.10 mg/mL indicated poor outcome at 3 months with a sensitivity of 70% and specificity of 82% (area under the curve: 0.815; 95% CI: 0.760–0.870; *P* < 0.0001) (Fig. S3).

When a logistic regression model was adjusted for female gender, atrial fibrillation, maximum axillary temperature, NIHSS at admission, volume of ischemic lesion, effective reperfusion, symptomatic HT or LA, and each biomarker separately, glutamate (OR: 1.01; CI 95%: 1.00–1.01, *P < *0.0001), TNF‐*α* (OR: 1.05; CI 95%: 1.01–1.09, *P* = 0.009), NSE (OR: 2.13; CI 95%: 1.50–2.90, *P < *0.0001), and S100‐*β* (OR: 5.86; CI 95%: 7.05–56.12, *P < *0.0001) were independently associated with poor outcome at 3 months in patients with IS through the connection with LA presence. Furthermore, the adverse outcome related to PDGF‐CC and sTWEAK was associated with mechanisms linked simultaneously to symptomatic HT and LA presence. Finally, serum sTWEAK concentrations at admission ≥ 6700 pg/mL were associated with an odds ratio of 13 for a poor outcome at 3 months (OR: 13.6; CI 95%: 8.2–22.6, *P < *0.0001).

## Discussion

HT is a dreaded complication that restricts the use of reperfusion treatment in individuals with IS. Mild hemorrhage (IH1, IH2) was usually associated with a good outcome, while severe symptomatic HT (PH1, PH2) acts as significant predictor of neurological deterioration and higher mortality.^4^ Previously published data suggest that there are negative side effects associated with rt‐PA. In particular, experimental stroke models have found neurotoxic side effects.^33^, ^34^ Furthermore, we have shown that administration of rt‐PA intravenously or intraarterially may be associated with a higher risk of functional deterioration in the first 3 months if reperfusion within the first 24 h does not happen.^35^ Therefore, predicting the development of early HT or exploring the relationship between biomarkers and HT subtypes would be helpful in guiding proper management of patients with IS, as well as to avoid adverse outcomes and poor prognosis.

In the present research work, clinical factors, neuroimaging variables and biomarkers associated to inflammation, endothelial and atrial dysfunction or BBB dysfunction were evaluated to find potential HT risk factors for patients with IS undergoing reperfusion treatments. Symptomatic HT incidence among patients with IS was 32% in our study and is comparable with the incidence in other studies, which is around 40%.^1^, ^2^, ^3^, ^4^, ^36^ Our data also indicated that IH1 subtype corresponded to an effective reperfusion and an improved functional outcome, but PH1 and PH2 subtypes were related with unfavorable outcome of patients at 3 months. Then, a rapid improvement ≤8 points in the NIHSS during the first 24 h might be a predictor of good outcome.

Among our patients with symptomatic HT, LA incidence was 75.4%. The presence and degree of LA correlated with a significant negative influence in IS. These results are in agreement with recent research, where different degrees of LA differentially influence functional outcomes at 3‐months follow‐up in patients with middle cerebral artery occlusion following intravenous thrombolysis.^12^

The results of our study showed that individuals with poor outcome had higher levels of sTWEAK at admission. By means of logistic regression models we determined that LA and symptomatic HT were interrelated variables. Therefore, we speculate the potential role of sTWEAK in the mechanisms associated to LA and HT. However, further preclinical studies are needed to elucidate these mechanisms. Moreover, two multivariate models with HT subtypes or LA grades noted that female gender, atrial fibrillation, NIHSS at admission, effective reperfusion, maximum axillary temperature, ischemic lesion volume, and C‐ reactive protein were independently associated with unfavorable outcome at 3 months for patients with IS.

Comparisons among different potential HT biomarkers concluded that biomarkers related to the BBB (MMP‐9, tight‐junction proteins, S100‐*β*, glial fibrillary acidic protein [GFAP], NSE) should be the most promising predictors of symptomatic HT, as well as that inflammation indicators (leukocyte, C‐reactive protein, TNF*α*, IL‐6, IL‐10, vascular adhesion protein‐1 [VAP‐1]) would be a correct choice for predicting delayed HT.^8^ Apart from inflammatory indicators, the more frequent HT biomarkers in our patients with unfavorable outcome were sTWEAK, glutamate, TNF*α*, IL‐6, NSE, S100‐*β*, and PDGF‐CC. It is interesting to note that all biomarkers used in our analysis were associated with HT severity and with the presence and degree of LA (except TNF‐*α*, NSE and S100‐*β*; and neuroserpin, respectively). Finally, by means of logistic regression models we could determinate that PDGF‐CC and sTWEAK were associated with mechanisms linked simultaneously to HT and LA presence. In particular, serum sTWEAK levels at admission ≥6700 pg/mL increased the odds by a factor of 13 for an unfavorable outcome at 3 months. Therefore, sTWEAK may be considered to play a role in the mechanisms associated with HT, as well as LA.

sTWEAK is a type II transmembrane glycoprotein of the TNF superfamily that acts by binding to a small transmembrane type I protein (Fn14). It has been demonstrated that sTWEAK involves the expression levels of cytokines, cell adhesion molecules, tight junction proteins, and MMPs in cultured endothelial cells. It also alters the permeability of an in vitro BBB model.^37^, ^38^, ^39^ These mechanisms could modulate the LA progression and encourage HT presence by increasing the bleeding. Thus, the possibility of blocking the activation of the sTWEAK‐Fn14 system could represent a diagnostic and a therapeutic option for patients with IS undergoing reperfusion treatments, for which further studies will be necessary.

Our study presents some weaknesses: First, we conducted a retrospective single‐center study with no preclinical data supporting the molecular mechanisms involved in the interactions of sTWEAK with symptomatic HT. Second, we studied sTWEAK and not the TWEAK‐Fn14 association, which could have more implications for the development of HT. Three, regarding the feasibility of the samples: glutamate and sTWEAK rate measurements were performed in 100% of individuals. However, IL‐6 in 96.1%, TNF*α* in 95%, MMP‐9 in 91.9%, S100‐*β* in 29.5%, NSE in 29.5%, neuroserpin in 30.7%, c‐Fn levels in 92%, ET‐1 in 20.9%, and PDGF‐CC in 28.6% of total patients. Four, HT biomarker measurements were not simultaneously performed and were made by different researchers, although the measurements were always blind to clinical and neuroradiological data and supervised by the same senior researchers. Five, we have not considered the different reperfusion treatments (intravenous or intraarterial fibrinolysis, thrombectomy, or both procedures) in the analysis. The strong points of this work are the unbiased screening of individuals, the high number of enrolled patients, and the large number of biomarkers assessed.

## Conclusion

In patients with IS undergoing reperfusion treatments, serum levels at admission of sTWEAK ≥6700 pg/mL were associated with an odds ratio of 13 for an adverse functional outcome at 3 months. The presence of LA, as well as its progression, is a neuroimaging factor strongly associated with symptomatic HT and unfavorable outcome. In this regard, sTWEAK may have a role in mechanisms associated with HT, as well as LA.

## Conflict of Interest

The authors report no conflicts of interest.

## Authors' Contributions

Conception and design of the study (PH, RIR, JC). Data acquisition and analysis (FC, AD‐C, TS, MPM). Clinical data acquisition and analysis (JC, MRY, JP, ILD). Handled funding and supervision (RIR, TS, JC, PH, AD‐C). Statistical analysis (PH and JC). Manuscript drafting (RIR, PH, PAG, JC). Critical revision for important intellectual content (MPM, TS, JC, FC). Supervision (JC, MRY, JP, ILD, PAG). All authors reviewed and approved the manuscript.

## Supporting information


**Figure S1.** Association between HT incidence and serum levels at admission of NSE, S100‐*β*, neuroserpin, PDGF‐CC, sTWEAK, ET‐1, MMP‐9, c‐Fn, IL‐6, TNF*α*, and glutamate.
**Figure S2.** Association between LA presence and serum levels at admission of NSE, S100‐*β*, neuroserpin, PDGF‐CC, sTWEAK, ET‐1, MMP‐9, c‐Fn, IL‐6, TNF*α*, and glutamate.
**Figure S3.** ROC curve to establish the sensitivity and specificity of (A) S100‐*β* and (B) sTWEAK serum levels to predict adverse functional outcome at 3 months in patients with IS undergoing reperfusion treatments.
