# Supplementary material for: The presence of leukoaraiosis enhances the association between sTWEAK and hemorrhagic transformation
Source: Ann Clin Transl Neurol. 2020 Oct 6;7(11):2103–14. doi: 10.1002/acn3.51171 (PMC7664267; doi:10.1002/acn3.51171)

**The presence of leukoaraiosis enhances the association between sTWEAK and hemorrhagic transformation**

Andrés da Silva PhD^1^, María Pérez-Mato PhD^2^, Manuel Rodríguez-Yáñez MD PhD^3^, Iria López-Dequidt MD PhD^3^, José M Pumar MD PhD ^4^, Paulo Ávila-Gómez BSc^1^, Tomás Sobrino PhD^1^, Francisco Campos PhD^1^, José Castillo MD PhD^1^, Pablo Hervella PhD^1*^, Ramón Iglesias-Rey PhD^1*^

^1^ Clinical Neurosciences Research Laboratory (LINC), Health Research Institute of Santiago de Compostela (IDIS), Santiago de Compostela, Spain

^2^Neuroscience and Cerebrovascular Research Laboratory, La Paz University Hospital, IdiPAZ, UAM, Paseo de la Castellana 261, 28046, Madrid, Spain

^3^Stroke Unit, Department of Neurology, Hospital Clínico Universitario, Santiago de Compostela, Spain

^4^Department of Neuroradiology, Hospital Clínico Universitario, Health Research Institute of Santiago de Compostela (IDIS), Santiago de Compostela, Spain

**Address for correspondence:**

Ramón Iglesias-Rey ([ramon.iglesias.rey@sergas.es](mailto:ramon.iglesias.rey@sergas.es))

Pablo Hervella ([pablo.hervella.lorenzo@sergas.es](mailto:pablo.hervella.lorenzo@sergas.es))

Hospital Clínico Universitario, Rúa Travesa da Choupana, s/n 15706 Santiago de Compostela, Spain. Telephone/ Fax number: +34 981951098/+34 981951098

**Supplementary Material**

**Figure S1:** Association between HT incidence and serum levels at admission of NSE, S100-β, neuroserpin, PDGF-CC, sTWEAK, ET-1, MMP-9, c-Fn, IL-6, TNFα, and glutamate.


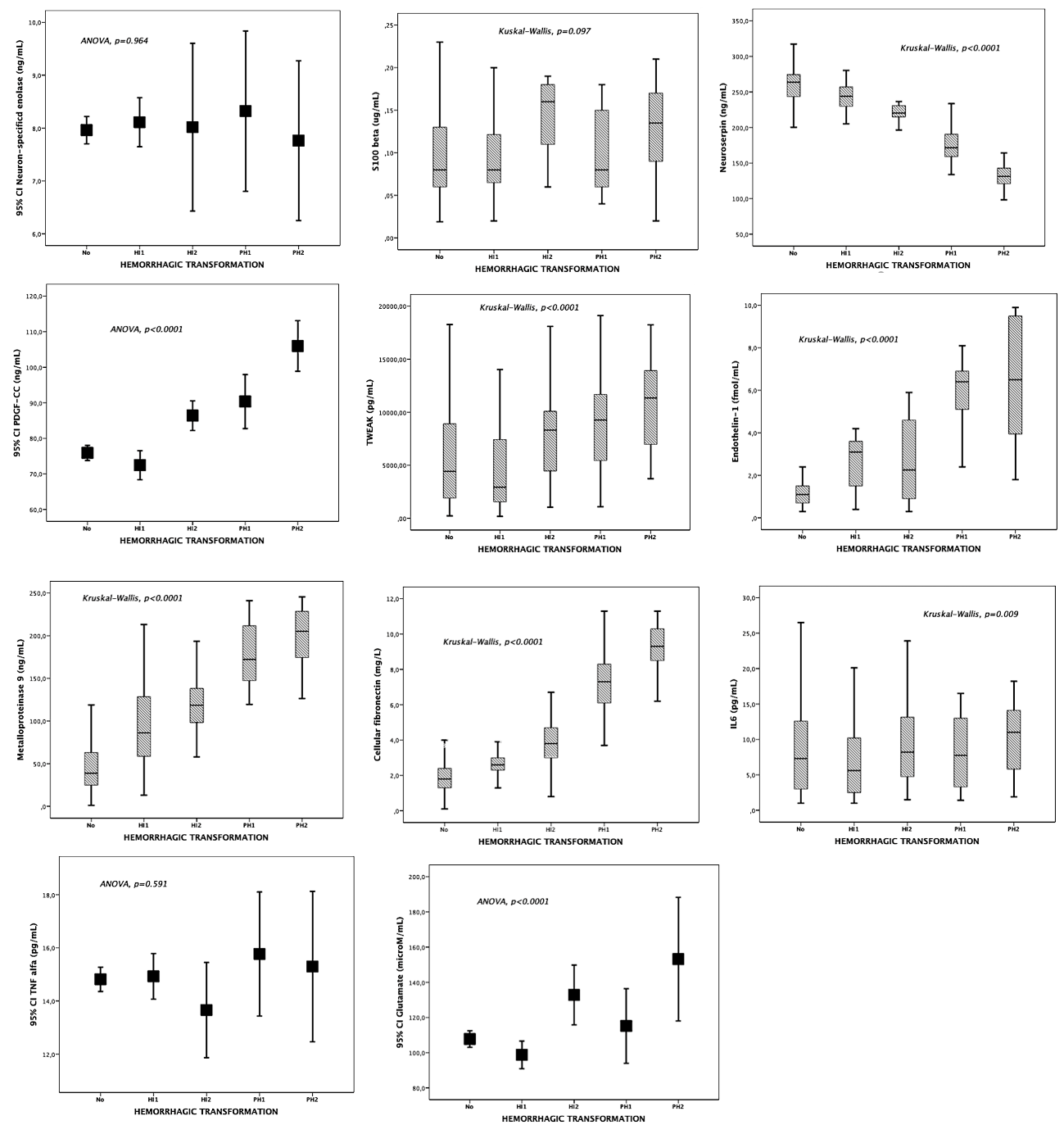


**Figure S2:** Association between LA presence and serum levels at admission of NSE, S100-β, neuroserpin, PDGF-CC, sTWEAK, ET-1, MMP-9, c-Fn, IL-6, TNFα, and glutamate.


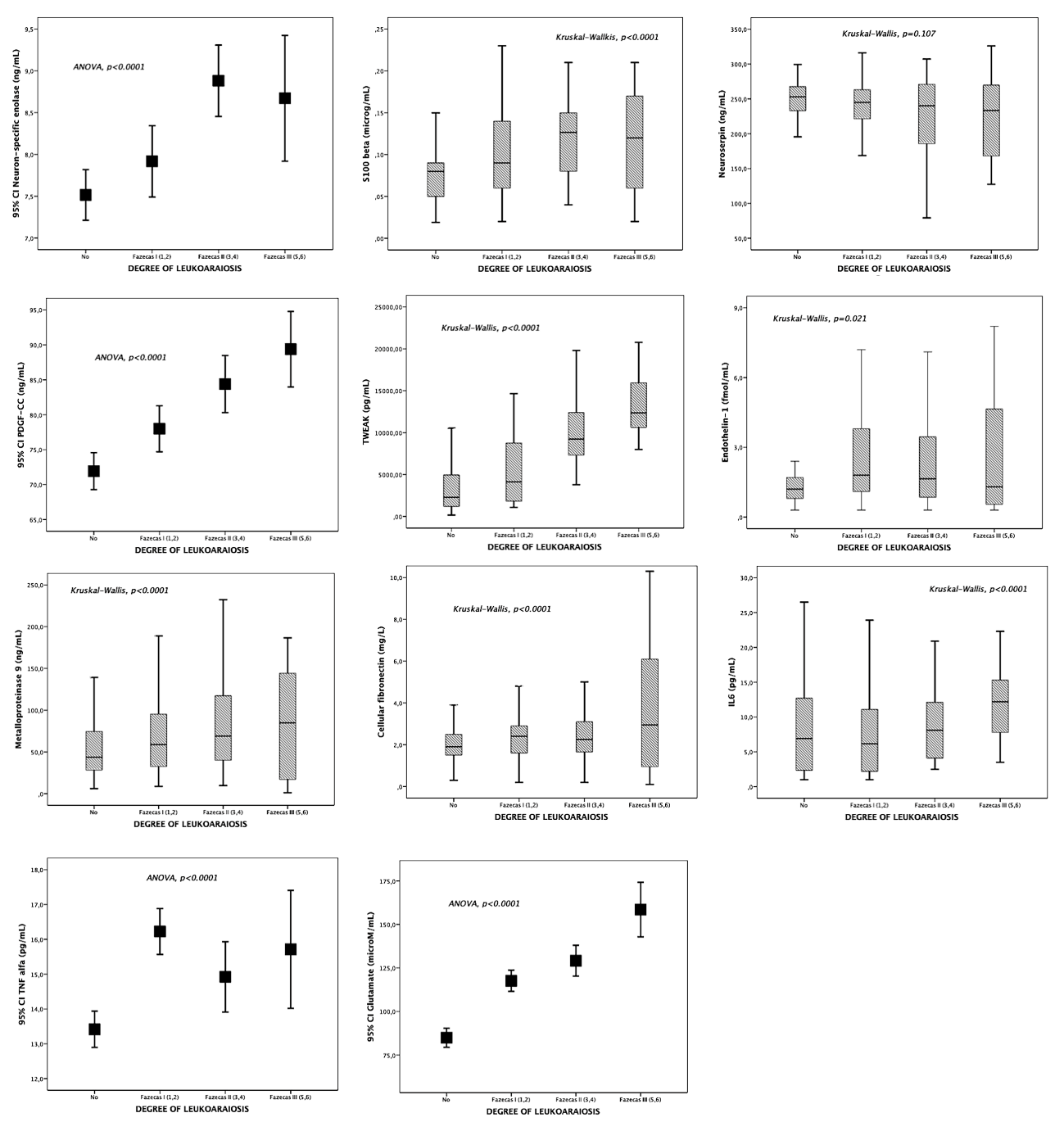


**Figure S3:** . ROC curve to stablish the sensitivity and specificity of **(A)** S100-β and **(B)** sTWEAK serum levels to predict adverse functional outcome at 3 months in patients with IS undergoing reperfusion treatments.


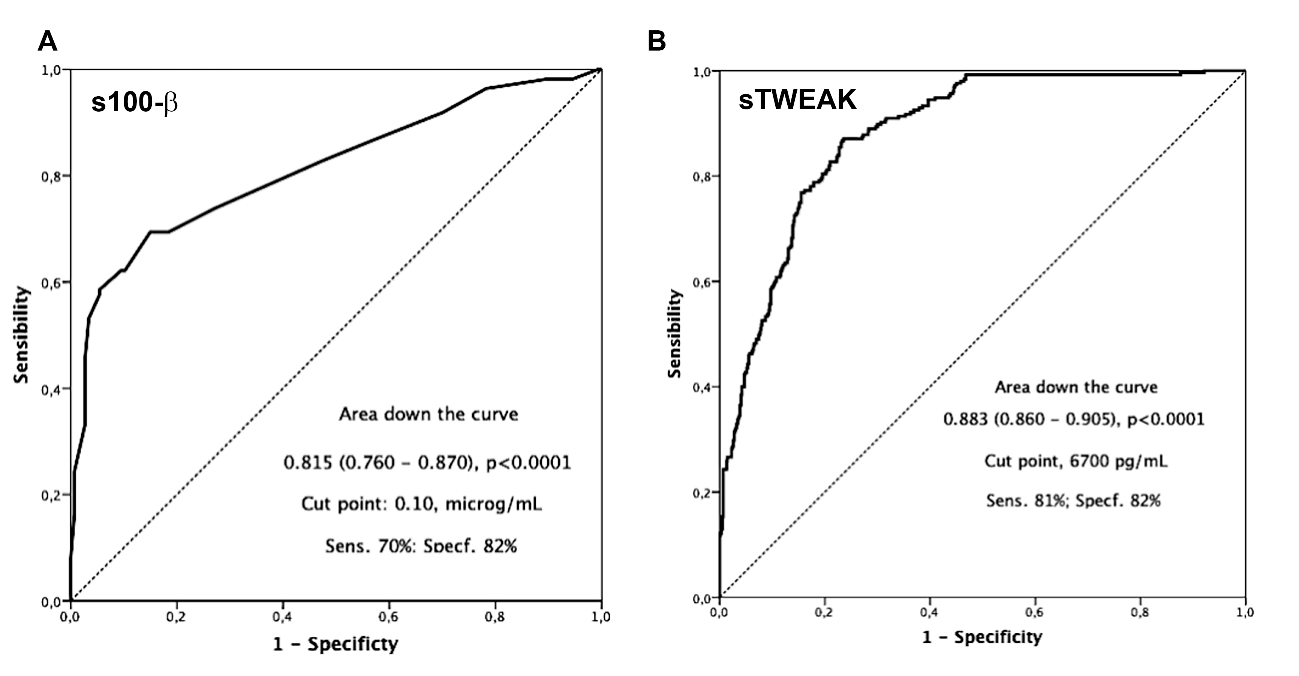

Supplement: Supplementary file 1 — Figure S1. Association between HT incidence and serum levels at admission of NSE, S100‐β, neuroserpin, PDGF‐CC, sTWEAK, ET‐1, MMP‐9, c‐Fn, IL‐6, TNFα, and glutamate. Figure S2. Association between LA presence and serum levels at admission of NSE, S100‐β, neuroserpin, PDGF‐CC, sTWEAK, ET‐1, MMP‐9, c‐Fn, IL‐6, TNFα, and glutamate. Figure S3. ROC curve to establish the sensitivity and specificity of (A) S100‐β and (B) sTWEAK serum levels to predict adverse functional outcome at 3 months in patients with IS undergoing reperfusion treatments. [file ACN3-7-2103-s001.docx]
